# DCLK1 isoforms and aberrant Notch signaling in the regulation of human and murine colitis

**DOI:** 10.1038/s41420-021-00526-9

**Published:** 2021-06-17

**Authors:** Badal C. Roy, Ishfaq Ahmed, Jason Stubbs, Jun Zhang, Thomas Attard, Seth Septer, Danny Welch, Shrikant Anant, Venkatesh Sampath, Shahid Umar

**Affiliations:** 1 Department of Surgery, University of Kansas Medical Center, Kansas City, KS USA; 2 Department of Internal Medicine, University of Kansas Medical Center, Kansas City, KS USA; 3 Children’s Mercy Hospital, Kansas City, MO USA; 4 Children’s Hospital, Aurora, CO USA; 5 Department of Cancer Biology, University of Kansas Medical Center, Kansas City, KS USA

**Keywords:** Infection, Bacterial infection

## Abstract

Alternative promoter usage generates long and short isoforms (DCLK1-L and DCLK1-S) of doublecortin-like kinase-1 (DCLK1). Tight control of Notch signaling is important to prevent and restitute inflammation in the intestine. Our aim was to investigate whether Notch1–DCLK1 axis regulates the mucosal immune responses to infection and whether this is phenocopied in human models of colitis. In the FFPE (formalin-fixed paraffin-embedded) sections prepared from the colons of ulcerative colitis (UC) and immune-mediated colitis (IRAEC) patients, expression of DCLK1 isoforms correlated positively with Notch1 and negatively with a transcriptional repressor, FoxD3 (Forkhead Box D3). DCLK1 protein staining in these sections was predominantly sub-epithelial (stromal) wherein DCLK1 co-localized with NICD, CD68, CD11c, and neutrophil elastase (NE). NE also co-stained with Citrullinated-H3 indicating the presence of neutrophil extracellular traps. In human neutrophils, elevated levels of DCLK1-S, CXCL-10, Ly6G, MPO, NE, and Notch1/2 in LPS-treated cells were inhibited when LPS was added in conjunction with Notch blocker dibenzazepine (DBZ; LPS + DBZ group). In CR-infected *Rag1*^*−/−*^ mice, higher levels of DCLK1 in the colonic crypts were inhibited when mice received DBZ for 10 days coincident with significant dysbiosis, barrier disruption, and colitis. Concurrently, DCLK1 immunoreactivity shifted toward the stroma in CR + DBZ mice with predominance of DCLK1-S that coincided with higher Notch1 levels. Upon antibiotic treatment, partial restoration of crypt DCLK1, reduction in MPO activity, and increased survival followed. When intestinal epithelial cell-specific Dclk1-knockout (*Dclk1*^*ΔIEC*^) or *Dclk1*^*ΔIEC*^*;Rag1*^*−/−*^ double knockout (DKO) mice were infected with CR and given a single dose of DBZ, they developed barrier defect and severe colitis with higher levels of stromal DCLK1-S, Ly6G, NE, and Notch1. We therefore propose that, by regulating the mucosal immune responses, the Notch–DCLK1 axis may be integral to the development of murine or human colitis.

## Introduction

Inflammatory bowel disease (IBD) is a polygenic disease. An abnormal immune response mounted toward the luminal microbiota in a genetically susceptible host may lead to the onset and perpetuation of the disease [1, 2]. Tuft cells in the intestine that exhibit strong expression of doublecortin-like kinase 1 (DCLK1) [3] interact closely with cells of the immune system [4–7] and their ablation impairs epithelial proliferation and tissue regeneration after injury [8]. DCLK1 exists in long and short isoforms (DCLK1-L and DCLK1-S) generated by two alternative promoter usage with important differences between the isoforms in both human and mouse tissues. During human neoplasia, hypermethylation of DCLK1-L appears to cause a predominant switch to the short isoform, which confers a more invasive tumor phenotype [9] suggesting that DCLK1 isoforms likely have distinct functions [10]. Indeed, DCLK1+ tuft cells have recently been identified as new players in colitis [11]. Yet, the mechanisms regulating the biology of the two isoforms in murine or human models of colitis have not been clearly elucidated.

Notch signaling plays a critical role in the maintenance of epithelial integrity by regulating the balance of secretory and absorptive cell lineages and also by facilitating epithelial cell proliferation. In murine models of colitis, Notch is activated in the inflamed mucosa to stimulate cellular proliferation and regeneration of the tissue [12]. A recently published study clearly demonstrated that inhibition of Notch signaling decreases DCLK1+ cells following radiation injury [13] suggesting that Notch–DCLK1 axis may be an important signaling mechanism for crypt regeneration. Despite recent advances, many fundamental questions remain about the biology of the DCLK1 isoforms and whether Notch–DCLK1 axis regulates the mucosal immune responses to infection.

Models of infectious colitis are crucial for defining the potentially pathogenic host responses to enteric bacteria. Infection with *Citrobacter rodentium* (CR), a noninvasive mouse pathogen that belongs to the group of attaching and effacing (A/E) bacteria, is one of the few available models of infectious colitis [14, 15]. CR infection is used to model several important human intestinal disorders, including Crohn’s disease, ulcerative colitis (UC), and more recently, colon tumorigenesis [16, 17]. Following CR infection, mice develop colitis [18], and this causes a pronounced dysbiosis that is characterized by an overgrowth of CR and a consequent reduction in the abundance and overall diversity of the resident microbiota [19]. CR has become a model microorganism to study how enteric A/E pathogens subvert normal host cell functions, such as innate immunity, intestinal cell shedding, and apoptosis, to colonize the host [14].

Employing this well-credentialed model of infectious colitis, we previously demonstrated that chronic inhibition of Notch signaling coupled with decreases in DCLK1+ cells result in severe inflammation, morbidity, and mortality in an outbred strain that otherwise exhibits a self-limiting disease [20, 21]. These studies, however, did not elaborate on the effect of DCLK1 deletion on mucosal immune responses that regulate the outcome of infectious colitis [10]. We therefore hypothesized that mice lacking epithelial DCLK1 when challenged with CR infection may be highly susceptible to epithelial Notch inhibition and develop severe inflammation and colitis. Using both murine and human models of colitis, we demonstrate that loss of epithelial but not stromal DCLK1 synergizes with stromal Notch1 to regulate inflammatory processes in the colon.

## Results

### Differential expression of DCLK1 isoforms correlate positively with Notch1 and negatively with Forkhead Box D3 (FoxD3) expression

**Fig. 1:**
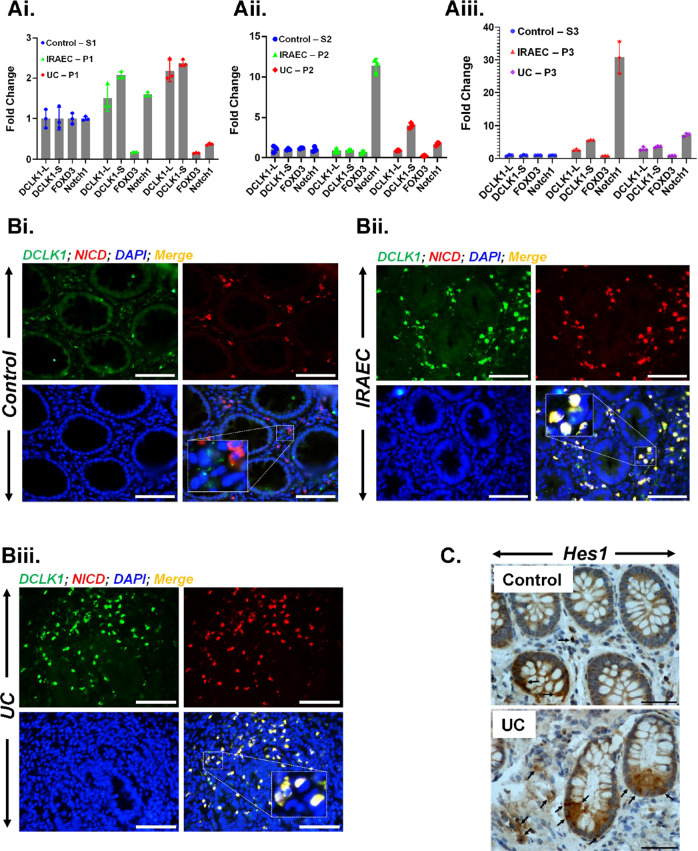
Correlation of DCLK1 isoforms with components of Notch signaling in the colons of UC and IRAEC patients. **A** Total RNA from the 10 μm FFPE sections was isolated followed by real-time RT-qPCR of the indicated markers. The values were normalized to GAPDH and data are presented as fold change. *P* values for **Ai** based on significance (IRAEC): 0.0029 (DCLK1-S), 0.0004 (FoxD3), 0.00016 (Notch1). *P* values for Ai based on significance (UC): 0.0042 (DCLK1-L), 0.0011 (DCLK1-S), 0.0003 (FoxD3), 0.00003 (Notch1). *P* values for **Aii** based on significance (IRAEC): 0.014 (FoxD3), 00004 (Notch1). *P* values for **Aii** based on significance (UC): 0.00013 (DCLK1-S), 0.000053 (FoxD3). *P* values for **Aiii** based on significance (IRAEC): 0.0015 (DCLK1-L), 0.000008 (DCLK1-S), 0.00044 (Notch1). *P* values for **Aiii** based on significance (UC): 0049 (DCLK1-L), 0.00019 (DCLK1-S), 0.043 (FoxD3), 0.000029 (Notch1); *n* = 3 independent experiments. **B** Paraffin-embedded sections from control or immune-mediated colitis (IRAEC) and ulcerative colitis (UC) patients were co-stained with antibodies for DCLK1 and NICD, respectively. Significant co-localization of DCLK1 with NICD was recorded in the sub-mucosal (stromal) regions in the colons of IRAEC (**Bii**) or UC (**Biii**) patients compared to control (**Bi**), shown as merged images (insets represent higher magnification of nuclear staining) while a subset of DCLK1+ cells did not co-localize with NICD (green staining). Scale bar = 100 μm; *n* = 2 independent experiments. **C** Representative images showing immunohistochemical staining of Hes-1, a downstream target of NICD. Scale bar, 80 μm; *n* = 2 independent experiments.

To understand the relationship between DCLK1 isoforms and Notch signaling, we isolated total RNA from the formalin-fixed paraffin-embedded (FFPE) sections of either control subjects or patients with UC or **immune**-related adverse events—colitis (IRAEC) and discovered distinct changes in the expression of DCLK1-L and DCLK1-S isoforms along with Notch1 and FoxD3, respectively. As is depicted in Fig. 1Ai–iii, while we observed elevated expression levels of both isoforms of DCLK1, DCLK1-S was particularly expressed at higher levels in both UC and IRAEC patients compared to control. These changes correlated with Notch1 upregulation, and a dramatic decrease in the expression of FoxD3 (Fig. 1A), a potent repressor of DCLK1-S expression in normal cells [22]. To corroborate changes in RNA levels with protein, we next stained 5 μm sections with antibodies against DCLK1 and Notch intracellular domain (NICD) that transduces Notch signaling initiated at the plasma membrane. As is depicted in Fig. 1B, compared to control (Fig. 1Bi), DCLK1 and NICD co-localization increased in UC and IRAEC samples wherein the protein staining was not only sub-epithelial (stromal) but predominantly nuclear consistent with NICD’s role in nuclear signaling (Fig. 1Bii, iii). Since DCLK1-L is a plasma membrane protein [23], these findings suggest that DCLK1-S may be predominantly co-localizing with NICD. As a corollary to this, membrane-bound DCLK1 did not co-localize with NICD (Fig. S1A). When sections were stained with antibody for Hes-1, the downstream target of Notch signaling, we observed significantly more staining for Hes-1 both within the crypts and the sub-epithelial of colons from UC patients compared to control (Fig. 1C) suggesting an active Notch signaling within the immune compartment.

**Fig. 2:**
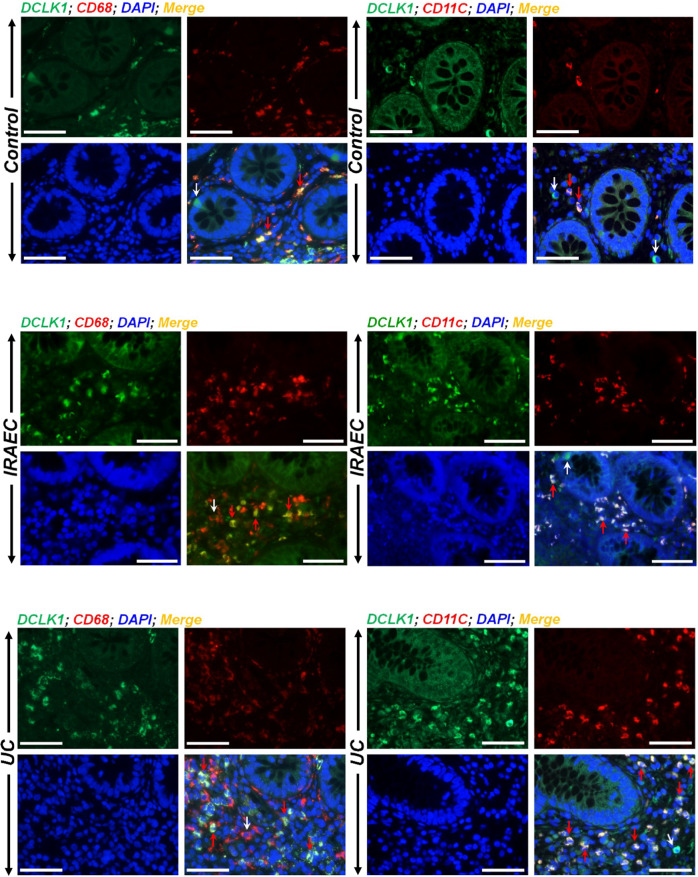
Immune cells associate with DCLK1 in the colons of UC and IRAEC patients. Paraffin-embedded sections from control or immune-mediated colitis (IRAEC) and ulcerative colitis (UC) patients were co-stained with antibodies for DCLK1/CD68 and DCLK1/CD11c, respectively. Nuclei were stained with DAPI (blue). Significant co-localization of DCLK1 with both CD68 and CD11c representing macrophages and dendritic cells, respectively, was recorded in the sub-mucosal (stromal) regions in the colons of IRAEC or UC patients compared to control (red arrows) while a subset of DCLK1+ cells did not co-localize with either cell type (white arrows). Scale bar = 100 μm; *n* = 2 independent experiments.

Next, we looked at DCLK1’s co-localization with markers of macrophages and dendritic cells (DCs) in the sections from IBD patients. Antibodies for epithelial cell adhesion molecule or CD45 were used to identify epithelial and immune cells, respectively (Fig. S1B). In control sections, we detected sub-epithelial DCLK1 co-localization with both CD68 and CD11c, respectively (Fig. 2A, B). In colonic sections from UC or IRAEC patients, there was weak staining recorded for epithelial DCLK1 while sub-epithelial regions exhibited significant co-localization of DCLK1 with both CD68 and CD11c, respectively (Fig. 2A, B). Not all DCLK1+ cells however, co-stained with markers of immune cells suggesting that these DCLK1+ cells may have specialized roles and may synergize with aberrant Notch signaling to influence stromal immune responses.

### CR infection increases the expression of DCLK1, a tuft cell marker, in the colons of immune-incompetent mice

**Fig. 3:**
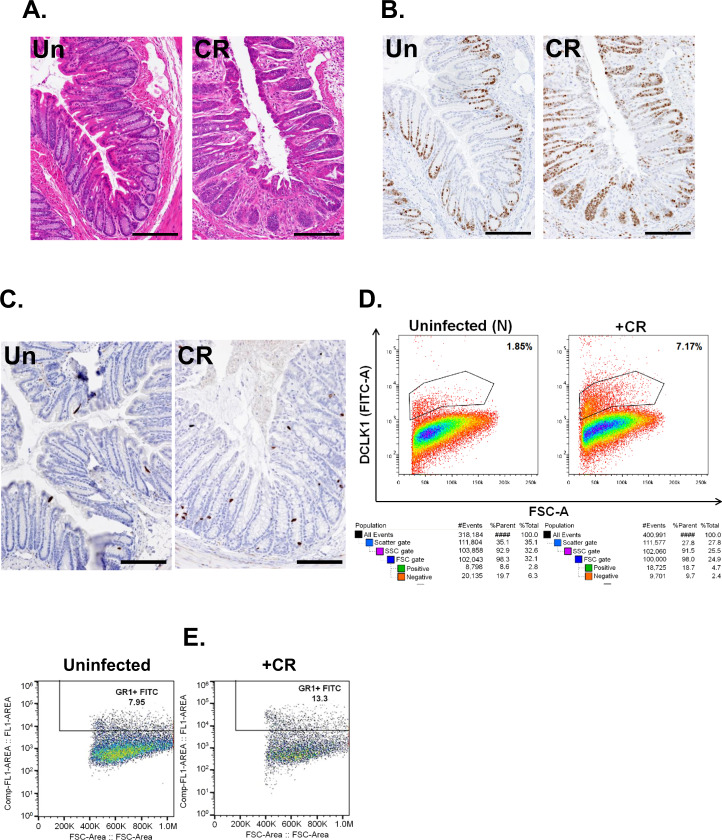
*Citrobacter rodentium* (CR) induced changes in gross morphology and DCLK1 expression. Representative H&E (**A**), Ki-67 (**B**), and DCLK1 (**C**) immunostaining of distal colons from uninfected (N) or CR-infected *Rag1*^*−/−*^ mice (scale bar 100 μm; 7–8 mice/group; *n* = 3 independent experiments). **D** Representative flow cytometry performed in crypt epithelial cells isolated from uninfected or CR-infected *Rag1*^-^^/^^-^ mouse colons for DCLK1 (lower panel represents the gating strategy). **E** Representative flow cytometry performed in the colons of uninfected or CR-infected wild-type C57BI/6j for Gr1(Ly6G).

To begin to understand the mechanistic basis of DCLK1–Notch axis in vivo, we utilized a well-established model of infectious colitis [18–20]. *Rag-1*^*−/−*^ mice compared to either FVB/N or C3H/HeN strains, when infected with CR, develop transient colitis and crypt hyperplasia [24]. In response to CR infection, the distal colons of *Rag1*^*−/−*^ mice had crypt hyperplasia, as was evidenced by hematoxylin and eosin (H&E) and Ki-67 staining (Fig. 3A, B). Intriguingly, we discovered a significant increase in DCLK1 staining in the distal colons of these mice compared to uninfected controls (Fig. 3C). This was confirmed through flow cytometry that revealed an ~4-fold (1.85 vs. 7.17%) increase in DCLK1+ cells in the colonic crypts isolated from CR-infected colon (Fig. 3D). In the immune-competent *Rag-1*^*+/+*^ mice, however, we did not see any increase in DCLK1 levels in response to CR infection compared to uninfected controls (2.57 vs. 2.44%) (data not shown). We next compared the extent of immune cell recruitment in the colons of CR-infected wild-type (WT) and *Rag-1*^*−/−*^ mice. As is depicted in Fig. 3E, Gr1(Ly6G)+ cells representing granulocytes increased from 7.95% in uninfected controls to 13.3% in CR-infected mouse colons from WT mice. In *Rag-1*^*−/−*^ mice, this increase was modest (8.57 vs. 10.1%; explained in 5C). Thus, CR infection upregulates DCLK1 in *Rag-1*^*−/−*^ vs. *Rag-1*^*+/+*^ controls in the colon that is not necessarily overtly inflamed.

### Blocking Notch signaling in vivo impairs CR’s proliferative capacity by promoting goblet cell hyperplasia

**Fig. 4:**
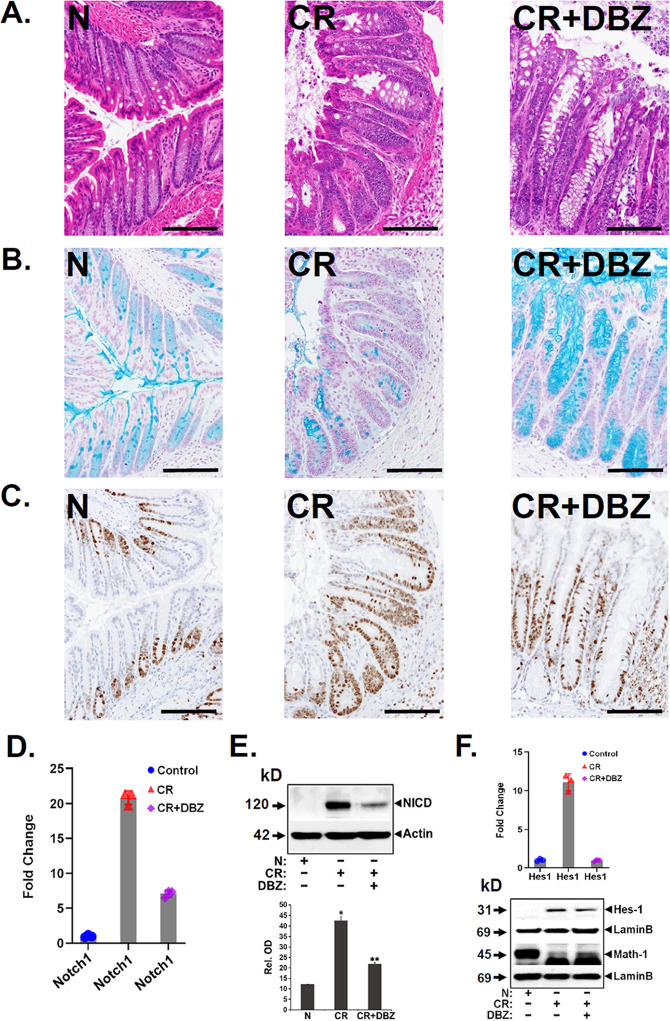
Effect of Notch blockade on signaling parameters. Representative H&E (**A**), Alcian blue (**B**), and Ki-67 (**C**) staining of uninfected (N), CR-infected (CR), or CR + DBZ-treated (CR + DBZ) *Rag1*^*−/−*^ mice (scale bar 100 μm; 7–8 mice/group; *n* = 3 independent experiments). **D** Total crypt RNA from the same group of mice was isolated and Notch1 expression was investigated via real-time RT-qPCR. The values were normalized to GAPDH and data presented as fold change. *P* values = 0.000004 (Notch1), 0.000091 (Hes1); *n* = 3 independent experiments. **E** Western blot analysis of NICD in the isolated crypts in the indicated group. β-Actin was used as the loading control. Lower panel. Densitometry showing relative abundance. *^,^**P < 0.05; *n* = 3 independent experiments. **F** Real-time RT-qPCR of Notch downstream target Hes-1 in the isolated crypts from the indicated groups. *P* values = 0.000091 (Hes1); *n* = 3 independent experiments. Lower panel showing western blot of Hes-1 and Math-1 in the crypts isolated from the indicated groups. Lamin B was used as the loading control (*n* = 3 independent experiments).

Combining CR infection with Notch signaling blockade results in disruption of the intestinal barrier and exacerbation of colitis [20]. We replicated this experiment in *Rag1*^*−/−*^ mice wherein CR infection coupled with Notch inhibition with a γ-secretase inhibitor dibenzazepine (DBZ) for 10 days revealed mucosal inflammation and crypt abscess along with goblet cell hyperplasia in the CR + DBZ-treated colons compared to CR alone (Fig. 4A, B). At the same time, the Ki-67 staining decreased significantly in the CR + DBZ-treated colons (Fig. 4C) compared to CR alone despite no change in crypt lengths. Since DBZ is a Notch blocker, we next investigated the effect of DBZ on components of Notch signaling. Of the four Notch receptors, we discovered a significant increase in Notch1 mRNA (Fig. 4D) and NICD generation (Fig. 4E) in the CR-infected crypts compared to uninfected controls that led to an increase in relative abundance for Hes-1 (Fig. 4F). CR + DBZ treatment decreased Notch1 mRNA, NICD, and Hes-1 levels (Fig. 4D–F, respectively). These data suggest that inhibition of epithelial Notch signaling that coincides with loss of crypt DCLK1 worsens CR-induced inflammation and suppresses proliferation in *Rag-1*^*−/−*^ mice.

### Notch inhibition impairs barrier function and promotes bacterial dissemination and neutrophil recruitment

**Fig. 5:**
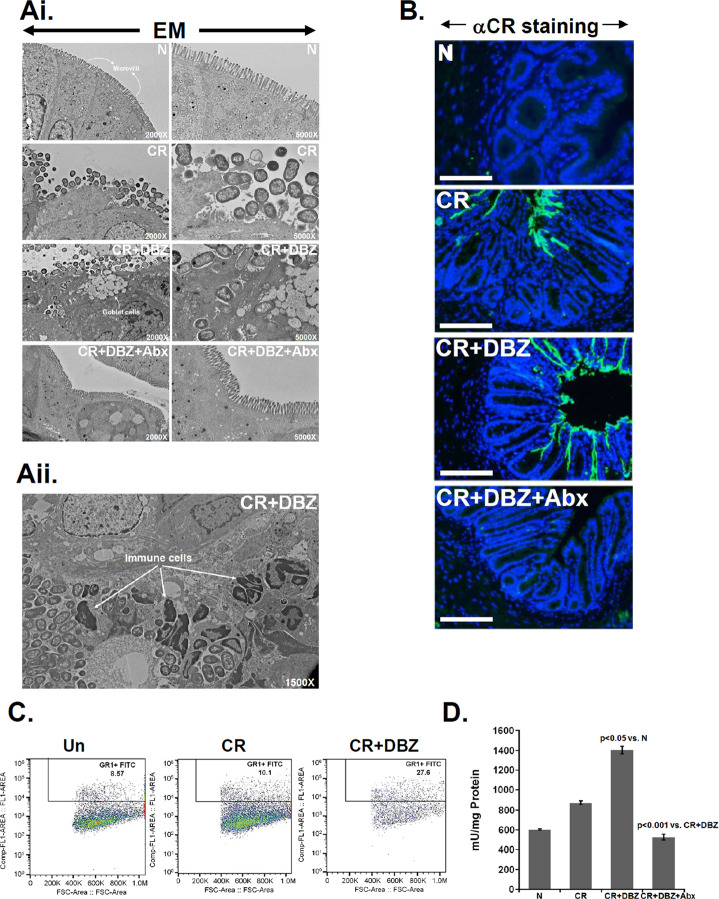
Effect of Notch blockade on bacterial colonization, neutrophil recruitment, and myeloperoxidase (MPO) activity. **Ai** Electron microscopy of distal colons isolated from uninfected (N), CR-infected (CR), CR + DBZ-treated (CR + DBZ), or CR + DBZ plus antibiotics (CR + DBZ + Abx)-treated *Rag-1−/−* mice. Loss of microvilli structure in response to CR infection was exacerbated following Notch inhibition in the CR + DBZ group with clear bacterial invasion into the sub-mucosa. Antibiotics (Abx) treatment restored the microvilli. **Aii** represents the EM of distal colon from the CR + DBZ group showing interaction of bacteria with immune cells (6 mice/group; *n* = 3 independent experiments). **B** Representative images showing bacterial staining by anti-CR antibody (αCR). **C** Flow cytometry for Gr1(Ly6G)+ cells was performed in the colon tissues of uninfected (N), CR-infected (CR), or CR + DBZ-treated (CR + DBZ) *Rag-1*^*−/−*^ mice. **D** MPO activity was measured in the colonic homogenates of the indicated groups using a Fluoro MPO Detection Kit (6 mice/group; *n* = 3 independent experiments).

To examine the consequence of Notch blockade in *Rag-1*^*−/−*^ mice on barrier function and bacterial dissemination, we performed fluorescein isothiocyanate (FITC) dextran assay and electron microscopy on colonic tissues in groups of mice indicated in Fig. 5. We observed a significant increase in serum FITC dextran levels in CR + DBZ colons compared to CR alone, indicating increases in paracellular permeability (Fig. S1C). CR infection disrupted the microvilli compared to uninfected controls (Fig. 5Ai). When CR infection was coupled with Notch inhibition by DBZ for 10 days, we discovered significant bacterial invasion toward the sub-epithelial regions some of which were associated with immune cells (Fig. 5Aii). To confirm whether these bacterial populations belonged to *Citrobacter*, we stained the sections with antibody to CR. As is depicted in Fig. 5B, sections from the uninfected colon were negative for CR, while significant CR staining was recorded in the lumen of CR-infected colons. In response to CR + DBZ, the CR staining extended into the crypts (Figs. 5B and S1D) and was associated with bacterial dissemination to the liver (Fig. S1E). These changes were associated with a significant increase in C-X-C chemokine motif ligand 1 (CXCL1)/keratinocyte (in mice) levels in both crypt and crypt-denuded lamina propria (CLP) in response to CR and CR + DBZ while changes in CXCL9/10 expression were less significant (Fig. S1F, G). When CR + DBZ mice were subjected to a cocktail of antibiotics, no such staining was observed (Fig. 5B). Since CXCL1 recruits neutrophils to the inflamed site [25], we isolated the CLP from the colons, and single cells were labeled with anti-Ly6G followed by flow cytometry. As is depicted in Fig. 5C, we discovered increases in Gr1(Ly6G)+ myeloid cells in the CR + DBZ group, which led to a significant increase in myeloperoxidase (MPO) activity compared to either CR or uninfected controls (Fig. 5D). In response to antibiotics, a decrease in MPO activity (Fig. 5D) and increased survival (data not shown) was observed in the CR + DBZ + Abx colons. Thus, barrier disruptions and bacterial dissemination accompany neutrophil recruitment/degranulation when CR infection is coupled with inhibition of epithelial Notch signaling in *Rag-1*^*−/−*^ mice.

### Chronic notch inhibition induces gut dysbiosis concomitant with alterations in crypt DCLK1

**Fig. 6:**
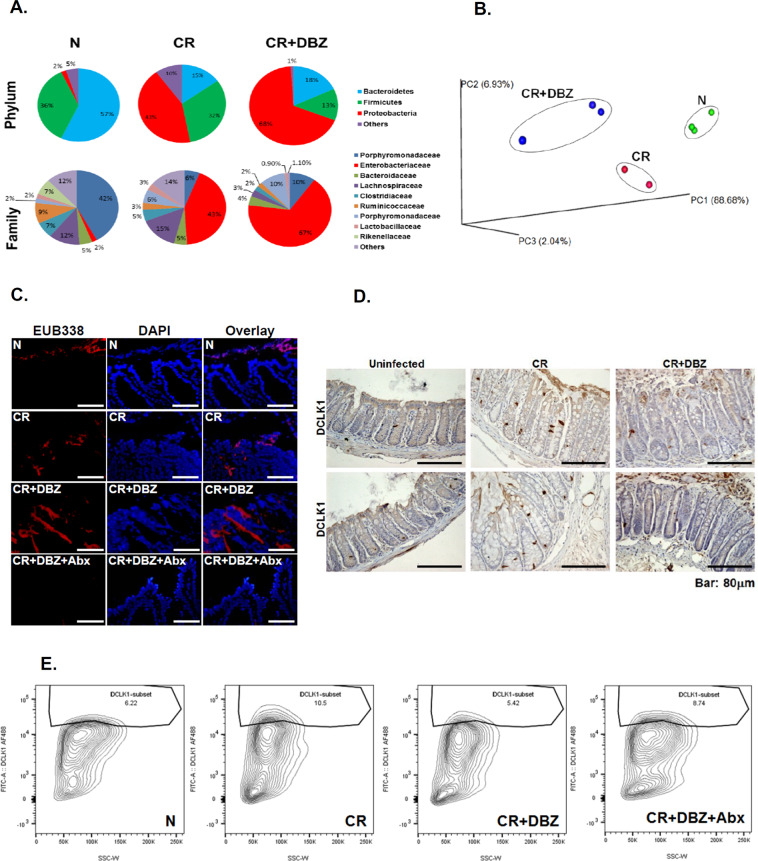
Microbial dysbiosis coincides with downregulation of crypt DCLK1 following Notch blockade. **A** Fecal samples from uninfected (N), CR-infected (CR), and CR + DBZ-treated mice were subjected to 16S rDNA sequencing and relative abundance of phyla and families were compared. Each chart represents the taxonomic composition in the indicated groups (*n* = 10 mice/group). **B** Principal coordinate analysis (PCoA) based on weighted UniFrac distances between bacterial communities. Please note significant separation of microbial communities in fecal samples from CR + DBZ mice when compared to either uninfected (N) or CR-infected (CR) mice (*P* < 0.05). **C** The bacteria in the colonic tissues of N, CR, CR + DBZ, and CR + DBZ + Abx-treated mice were detected by FISH using a general bacterial 16S probe (red, TexasRed-Eub338; bar = 100 μm; *n* = 3 independent experiments). DAPI was used as counterstain. **D** Representative images of DCLK1 staining in the distal colons of the indicated groups (*n* = 10 mice/group). **E** Flow cytometry showing percentages of DCLK1+ cells isolated from the groups of mice indicated (*n* = 10 mice/group).

In the stools collected from the CR-infected or CR + DBZ-treated groups, we observed significant dysbiosis with reduced levels of *Bacteroidetes* (15 and 18% compared to 57%) and *Firmicutes* (32 and 13% compared to 36%) accompanied by dramatic increases in *Proteobacteria* phyla (43 and 68% compared to 2%) (Fig. 6A). In particular, we discovered the *Enterobacteriaceae* family to be overrepresented in the CR + DBZ group (Fig. 6A). Principal coordinate analysis revealed a significant separation of microbial communities in fecal samples from CR + DBZ mice when compared to either uninfected or CR-infected mice (*P* value = 0.001; Fig. 6B). Interestingly, a controlled regimen of antibiotics cocktail (CR + DBZ + Abx) mitigated dysbiosis in CR + DBZ-treated *Rag1*^*−/−*^ mice, with increases in *Bacteroidetes* (31 vs. 16%) and *Firmicutes* (26 vs. 12%) and decreases in *Proteobacteria* (41 vs. 72%) (Fig. S2A). We next performed fluorescence in situ hybridization (ISH) analysis using a ubiquitous eubacterial probe EUB338. Results revealed only a small number of bacteria invading the colonic crypts in the CR group (Fig. 6C). In the CR + DBZ group, increases in bacterial invasion into the crypts were significantly reduced in the CR + DBZ + Abx group (Fig. 6C). Immunohistochemistry performed on tissue sections prepared from CR-infected mouse colon revealed a significant increase in DCLK1 staining compared to uninfected control (Fig. 6D) consistent with data shown in Fig. 3C, D. In the CR + DBZ group, significant reduction in DCLK1 expression was observed in the colonic crypts (Fig. 6D) as was also confirmed via flow sorting wherein the number of DCLK1+ cells in the crypts decreased from 10.5% in the CR group to 5.42% in the CR + DBZ group (Fig. 6E). In addition to DCLK1, CR infection also promoted Lgr5 expression, and the expression was attenuated in the CR + DBZ group (Fig. S2B). Thus, a loss of both crypt DCLK1-L and Lgr5 in the CR + DBZ group suggests that the mucosal regenerative capacity may be significantly compromised [26]. Interestingly, antibiotic treatment led to a decrease in *Proteobacteria* while both *Firmicutes* and *Bacteroidetes* showed an improvement, and these changes correlated with partial restoration of DCLK1+ cells compared to the CR + DBZ group (Fig. 6E). Thus, loss of crypt DCLK1 following inhibition of epithelial Notch signaling coincides with bacterial dysbiosis and severity of colitis.

### Crypt vs. stromal DCLK1 and changes in immune profile in response to CR infection and/or acute Notch inhibition

**Fig. 7:**
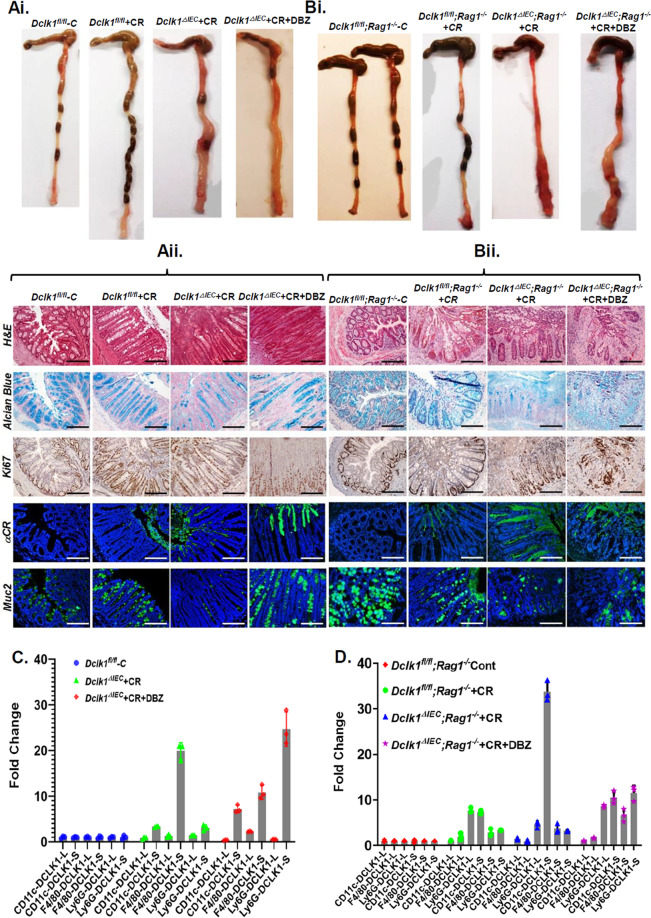
Effect of DCLK1 deletion on mucosal inflammation and colitis. Control (floxed) or intestinal epithelial cell-specific DCLK1-KO (*DCLK1*^Δ*IEC*^) or *DCLK1*^Δ *IEC*^*;Rag-1*^*−/−*^ double KO (DKO) mice were infected with CR and given single doses of DBZ. **Ai**, **Bi** Gross thickening and ulceration of the colon in the two transgenic lines. **Aii**, **Bii** Representative histology (H&E) or immunostaining in the paraffin-embedded sections prepared from the distal colons of the indicated mouse groups. Alcian blue and Ki-67 labeling represents goblet and proliferating cells, respectively. Anti-CR- and α-Muc2-stained *Citrobacter rodentium* and Muc-2, respectively (*n* = 6 mice/group; Scale bar: 100 μm). **C**, **D** Immune cells were FACS-sorted with antibodies against CD11c, F4/80, and Ly6G from the crypt-denuded lamina propria of the indicated groups of mice and the expression of the two isoforms of DCLK1 were measured by RT-qPCR. The values were normalized to GAPDH, and data are presented as fold change. *P* values for **C** based on significance: 0.000033 (CD11c: DCLK1-S), 0.000057 (F4/80: DCLK1-S), 0.013 (Ly6G: DCLK1-L), 0.0081 (Ly6G: DCLK1-S). *P* values for **D** based on significance: 0.000035 (CD11c: DCLK1-S), 0.016 (F4/80: DCLK1-S), <0.000001 (Ly6G: DCLK1-S); *n* = 3 independent experiments.

To investigate whether the expression of DCLK1 is critical in maintaining crypt integrity, we bred *DCLK1*^*fl/fl*^ mice with *CDX2-Cre/ERT2* to generate *DCLK1*^*ΔIEC*^ mice upon tamoxifen-induced *Cre* recombination (Fig. S3A). Since DCLK1 was significantly overexpressed in *Rag-1*^*−/−*^ mice in response to CR infection, we further bred *DCLK1*^*fl/fl*^*;CDX2-Cre/ER*^*T2*^ mice with *Rag1*^*−/−*^ to generate double knockout (DKO) mice to see whether the DKOs are more susceptible to infectious colitis. Figure S3B–E depict the genotypes of various crosses and a representative image of the DCLK1 staining in *DCLK1*^*fl/fl*^ and *DCLK1*^*ΔIEC*^ mice, respectively. In response to CR infection, both *DCLK1*^*ΔIEC*^ and DKO mice had significant gross thickening of the colon (Fig. 7Ai, Bi), elongated crypts showing elevated Ki-67 labeling, and goblet cell hypoplasia associated with loss of Muc-2 (Fig. 7Aii, Bii). We also detected significant CR staining in the lumen of CR-infected colons compared to the uninfected colon with some penetration into the crypts. When CR-infected mice were given a single dose of DBZ, more severe phenotypes were observed (Fig. 7Aii, Bii) that paralleled changes recorded in *Rag1*^*−/−*^ mice after 10 consecutive days of DBZ (see Figs. 6 and S1) indicating that loss of crypt DCLK1 severely impacts the disease process. In the absence of CR infection, however, DBZ alone did not have any associated pathology (data not shown) [19]. Interestingly, loss of crypt DCLK1 did not impact Lgr5 expression in CR-infected crypts (Fig. S4A); CR + DBZ treatment, however, led to a severe reduction in Lgr5 expression (Fig. S4A) as was reported elsewhere (see Fig. S2B). This was further corroborated in an organoid assay wherein the CR + DBZ group exhibited degeneration of the enteroids compared to the CR group (Fig. S4B).

Despite the loss of crypt DCLK1 in *DCLK1*^*ΔIEC*^ mice, we discovered stromal presence of DCLK1+ cells in CD11c+;MHCII+ DCs, F4/80+;MHCII+ macrophages, and Gr-1(Ly6G)+ neutrophils wherein there was a predominance of DCLK1-S in the sorted cells, particularly in Ly6G+ neutrophils (Fig. 7C). Interestingly, in vitro studies in cultured bone marrow-derived cells (BMDCs; Fig. S5A) only detected DCLK1-S in CR-infected cells, and the levels declined in response to CR + DBZ (Fig. S5B). In CLP, although we detected both the isoforms, only the DCLK1-S levels were induced by CR infection with an ~40% reduction in the levels after CR + DBZ treatment (Fig. S5B) paralleling those recorded in vivo (see Fig. 6). Since stromal DCLK1-L was not affected by either treatment, these findings suggest that DCLK1-S may be the target of Notch dysregulation within the stromal region. In DKO mice, DCLK1-S was consistently upregulated in CD11c+ and Ly6G+ cells (Fig. 7D). Intriguingly, we also observed the expression of DCLK1-L isoform (Fig. 7D), particularly in the Ly6G+ cells in the CR group, and its expression was not affected by DBZ (Fig. 7D). To see whether the two isoforms can also be regulated by proinflammatory cytokines, we next performed an in vitro assay wherein we observed interleukin-6-induced increases in both DCLK1-L and DCLK1-S promoter reporter activities, respectively (Fig. S5C). Taken together, *CDX2-Cre*-dependent KO of epithelial DCLK1 does not preclude accumulation of the two isoforms within the sub-epithelial region at least at the RNA level and that this may be influenced by immune cell recruitment.

**Fig. 8:**
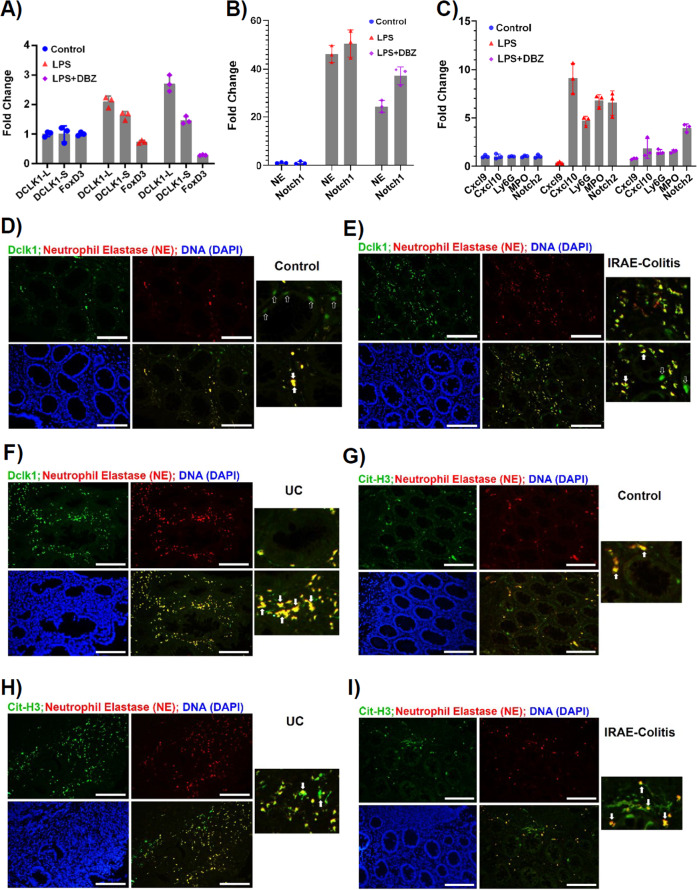
Evidence of neutrophil extracellular trap formation in vivo. **A**–**C** Human neutrophils were cultured in the presence or absence of LPS or LPS + DBZ for 8 h and the expression of indicated markers was determined by RT-qPCR and the data are presented as fold change after normalizing values to GAPDH. *P* values for **A** based on significance (LPS vs. control): 0.000734 (DCLK1-L), 0.023915 (DCLK1-S), 0.004097 (FoxD3); LPS + DBZ vs. LPS: 0.000525 (DCLK1-L), 0.057920 (DCLK1-S), 0.000035 (FoxD3). *P* values for **B** based on significance (LPS vs. Control): 0.000024 (NE), 0.000115 (Notch1); LPS + DBZ: 0.000083 (NE), 0.000075 (Notch1). *P* values for **C** based on significance (LPS vs. Control): 0.003059 (CXCL9), 0.000883 (CXCL10); LPS + DBZ vs. LPS: 0.007031 (MPO), 0.000315 (Notch2). **D**–**I** Paraffin-embedded sections from control or UC and immune-related adverse event—colitis (IRAEC) patients were co-stained with antibodies for DCLK1/NE or NE/Cit-H3, respectively. Solid arrows provide evidence of co-localization; open arrows point to DCLK1+ cells that did not co-localize with NE (Scale bar = 150 μm; *n* = 2 independent experiments).

Since Ly6G+ neutrophils had the highest levels of DCLK1-S, we next corroborated these findings in vitro wherein we treated human neutrophils with lipopolysaccharide (LPS) and DBZ and analyzed the expression profiles of the two DCLK1 isoforms and other relevant genes. As is depicted in Fig. 8A, LPS significantly increased both DCLK1-L and DCLK1-S isoforms along with 51- and 76-fold increases in neutrophil elastase (NE) and Notch1, respectively (Fig. 8B). In addition, we observed significant increases in CXCL10, Ly6G, MPO, and Notch2 in response to LPS (Fig. 8C). When LPS was combined with DBZ, DCLK1-L expression was not affected by DBZ (Fig. 8A). DCLK1-S levels, however, were mildly reduced upon DBZ treatment along with a reduction in NE, Notch1 and 2, CXCL10, Ly6G, and MPO expression, respectively (Fig. 8A–C) suggesting that these genes may be regulated by Notch signaling. In contrast, we saw significant downregulation of FoxD3 in LPS-treated samples and the expression was further reduced in the LPS + DBZ group (Fig. 8A). These results are in complete contrast to our findings in colonic crypts wherein we showed that membrane-bound DCLK1 is reduced upon DBZ treatment (Fig. 6D, E) suggesting that the two isoforms may be regulated differently in the epithelial vs. non-epithelial/stromal compartments. Since neutrophils besides providing a front line of defense against bacterial infection also act as mediators of inflammation [27], we next studied co-localization of NE with DCLK1 and Citrullinated-H3 (Cit-H3) to detect neutrophil extracellular trap (NET) formation in the FFPE sections. As is depicted in Fig. 8D–F, compared to control, we saw significant co-localization of NE with DCLK1 in both UC and IRAEC. NE also co-localized with Cit-H3 in both UC and IRAEC (Fig. 8G, H) consistent with neutrophilic inflammation seen in immune-mediated colitis patients [28]. These results provide an insight into which neutrophils’ recruitment into the colons may contribute toward tissue damage and severity of colitis.

## Discussion

Tuft cells interact closely with cells of the immune system and expand during chronic inflammation [29]. Yet, neither their biology nor the mechanism of their action is clearly understood. Recent studies have speculated that these cells, marked by DCLK1, may serve as a source of Notch signaling for Lgr5+ stem cells following removal of Paneth cells [30]; yet, the signaling mechanisms remain underexplored.

Employing human and murine models of colitis, we discovered that the two isoforms of DCLK1 are expressed differently in the epithelial vs. sub-epithelial regions of the inflamed mucosa. We also provide evidence that microbial imbalance induced by an enteric pathogen synergizes with loss of crypt DCLK1 and Notch dysregulation to promote deviant or aberrant immune activation that accompanies development of colitis. In response to CR infection, there was an expansion of the DCLK1+ cells reminiscent of tuft cell hyperplasia seen in the small intestine in response to parasitic infection [3, 5]. The increase in crypt DCLK1 was significantly inhibited when CR-infected mice were given ten doses of Notch blocker DBZ and coincided with microbial dysbiosis, barrier disruption, and release of proinflammatory mediators suggesting that Notch signaling is a critical intermediary pathway in gut dysbiosis regulation of DCLK1 expression. This is consistent with a recent report wherein DCLK1 was shown to mitigate mucosal barrier dysfunction by promoting intestinal repair to counter chronic inflammation [11]. Moreover, the observation that both *DCLK1*^*ΔIEC*^ mice and DKOs became hypersensitive to single doses of DBZ further portends that tight control of Notch signaling is important to prevent or restitute inflammation in the gut.

Notch signaling has recently been implicated in the control of several innate cell populations [31]. In the current study, we observed various immune cell populations, including macrophages, DCs, and particularly neutrophils to express DCLK1-S isoform. In vitro studies in neutrophils further provided evidence that LPS-induced increases in DCLK1-S isoform but not the DCLK1-L variant was reduced in cells treated with LPS + DBZ. This is in contrast to the downregulation of DCLK1 in the crypt epithelial cells isolated from CR + DBZ-treated mice (see Fig. 6D, E). Interestingly, expression of several genes, including FoxD3, CXCL10, Ly6G, MPO, NE, and Notch1/2, were reduced when the Notch pathway was blocked, thereby indicating that they may be involved in DCLK1-S regulation. FoxD3 in particular has recently been reported to inhibit the transcriptional activity of the DCLK1’s β-promoter thereby repressing DCLK1-S expression [22]. While available data indicate that loss of FoxD3 due to epigenetic silencing promotes overexpression of the DCLK1-S variant in human colon cancer cells [22], no such data exist in non-cancer models. Thus, although a limitation of our study, it is nonetheless tempting to speculate that increases in DCLK1-S seen in the current study may be a result of epigenetic silencing of the FoxD3 gene.

Neutrophils play both protective and detrimental roles in mucosal homeostasis. Antimicrobial activity of neutrophils is mediated by phagocytosis, the release of reactive oxygen species, and lytic enzymes, as well as the formation of NETs composed of DNA, histones, and granular proteins, such as NE [32]. This is consistent with recent reports describing excessive NE activity associated with tissue damage in a murine model of colitis [33] and intraepithelial infiltration of human NE (HNE)+ cells in UC patients [34]. Along these lines, NE not only co-localized with DCLK1 in the colons of both UC and IRAEC patients but also exhibited co-localization with Cit-H3 consistent with neutrophilic inflammation seen particularly during IRAEC [35]. Whether Notch signaling is definitively involved in NET formation in these colons remains to be determined. Our discovery that DCLK1 co-localized with NICD in the colons of both UC and IRAEC patients does, however, provide a clue to the possibility that it may facilitate NET formation in a Notch-dependent manner to regulate mucosal inflammation. A recent study supporting this notion elegantly demonstrated that macrophage-specific deletion of RBP-J, a transcription factor that signals through Notch receptors, leads to impaired innate and adaptive immune responses, thereby impacting bacterial clearance [36].

Since gut bacteria are critical for the development of colitis as their depletion leads to attenuation of the inflammatory processes, we acknowledge the limitation of our study to fully implicate gut microbiome changes associated with aberrant Notch signaling in either the development or progression of colitis in transgenic mouse lines. To this end, we did, however, see moderate increase in DCLK1+ cells in *Rag-1*^*−/−*^ mice treated with antibiotics (see Fig. 6E) suggesting that restoring crypt DCLK1 may help attenuate infectious colitis. Our findings also indicate that stromal Notch activity fueled by immune cell recruitment, particularly neutrophils, may synergize with DCLK1-S to regulate the severity of infectious colitis. We corroborated some of these findings in the colons of UC and IRAEC patients to further establish the translational nature of our study. In conclusion, while DCLK1 has mostly been studied as a marker of tuft cells [3–8], our findings indicate that DCLK1 isoforms likely have distinct functions, and the expression of these isoforms may occur in different cellular compartments that may not all represent tuft cells. Thus, while more studies are needed to characterize the role of DCLK1 isoforms as immune sentinel to connect intestinal lumen with immune cells in the underlying tissue, we posit that loss of epithelial but not stromal DCLK1 synergizes with stromal Notch1 to regulate inflammatory processes in the colon.

## Materials/subjects and methods

### Mouse models and procedures

This study was carried out in strict accordance with the recommendations in the Guide for the Care and Use of Laboratory Animals of the National Institutes of Health. All protocols were approved by the University of Kansas Medical Center Animal Care and Use Committee. All efforts were made to minimize suffering. WT C57Bl/6j and B6.129S7-*Rag1tm1Mom*/J (*Rag-1*^*−/−*^) mice were procured from Jackson Laboratory, Bar Harbor, ME, USA. The DCLK1-floxed mice (*DCLK1tm.2Jgg*; stock#013170) surrounding exon 3 of the *DCLK1* gene were crossed with *CDX2-Cre/ERT2* (B6.Cg-Tg; CDX2-Cre/ERT2)752ERF/J) mice to obtain colon-specific KO of DCLK1 (*DCLK1*^*ΔIEC*^). Further, we bred *DCLK1fl/fl;CDX2-Cre* mice with *Rag1*^−/−^ to generate *DCLK1fl/fl;CDX2-Cre;Rag1*^*−/−*^ DKO mice. The progeny was genotyped for both the presence of *floxed* site and *CDX2-Cre* recombinase by tail-tip biopsies using the set of primers described in Table 1. WT littermates or transgenic mice (C57BL/6J background; 5–6 weeks old) received sterile culture medium or were infected by oral inoculation with a 16-h culture of CR (biotype 4280, ATCC, 10^8^ colony-forming units), identified as pink colonies on MacConkey agar, as previously described [17–21, 27, 28] coupled with tamoxifen injection (intraperitoneally (i.p.; (75 mg/Kg; 2 doses, alternately at postinfection days 3 and 5) for conditional gene KO. To block Notch signaling in vivo, WT, *Rag1*^*−/−*^ mice (10 doses; beginning at 2 days post-CR infection), or transgenic lines (single dose; postinfection day 6) were treated with cell-permeable γ-secretase inhibitor, DBZ (EMD Chemicals, Inc.) [37] i.p. at 10 μmol/Kg of body weight suspended in 0.5% (wt/vol) hydroxypropyl-methylcellulose and 0.1% Tween-80 in water (wt./vol.).

**Table 1:** List of primers used in the study.

Genes	Forward primers	Reverse primers
Human Dclk1-L	aaacggctcattcctttgag	agtcctgaaggcacatcacc
Mouse Dclk1-S	gtcagccttacgcaggaaaa	tgggaagcagttggattagc
Human Dclk1-S	aggcatctgctgatgaatcc	tctcagcactaagccaagca
Mouse NE	gcactggcctcagagattgt	cagaaatgacctccacgcct
Human NE	aacgtctgcactctcgtgag	gaaggaggcaattccgtgga
Mouse FoxD3	tgcagctacagctcaacacc	tgttctcgatgctgaacgac
Human FoxD3	caaccgcttcccctactaca	gggatcttgacgaagcagtc
Mouse Cxcl-1	cttgaaggtgttgccctcag	tggggacaccttttagcatc
Mouse Cxcl-9	acggagatcaaacctgccta	tttttccccctcttttgct
Human Cxcl-9	gagtgcaaggaaccccagta	ttggggcaaattgtttaagg
Mouse Cxcl-10	gctgcaactgcatccatatc	cgtggcaatgatctcaacac
Human Cxcl-10	ccccacgttttctgagacat	aaggcagcaaatcagaatgg
Mouse MPO	tacccccgagactttgtcag	atagcacaggaaggccaatg
Human MPO	aggacaaataccgcaccatc	gaagagagaagccgtcctca
Mouse Notch2	cctgaacgggcagtacattt	gcgtagcccttcagacactc
Human Notch2	tatatttgcacctgcccaca	ttttcctgcatgctcacaag
Mouse GAPDH	aactttggcattgtggaagg	acacattgggggtaggaaca
Human GAPDH	aggctggggctcatttgcagg	tgaccttggccaggggtgct
For genotyping		
Cdx2 transgene	acatgtccatcaggttcttgc	aggagccagcggagcac
Dclk1	cttcccactgatatgttcattc (Mutant)	agtgagatggtttacaggcaag (Common)
Rag1	tggatgtggaatgtgtgcgag (Mutant)	cattccatcgcaagactcct (Common)

### Bacterial DNA extraction and microbiota analysis using genomic DNA

Total genomic bacterial DNA from fresh feces was extracted using the QIAmp DNA Stool Kit (Qiagen, Valencia, CA). DNA solutions were stored at −80 °C until further processing. Using gDNA, the V4 region of the 16S rRNA encoding gene was amplified with barcoded universal bacterial primers followed by sequencing on Ion Torrent platform [19]. The resulting raw sequence files (.fastq.gz) are being submitted to the NCBI Sequence Read Archive (SRA) database. The raw sequences were analyzed using Quantitative Insights Into Microbial Ecology (QIIME)-2 pipeline [38].

### EUB338 staining and ISH

For EUB338 staining, paraffin sections were dewaxed and rehydrated in an ethanol to water gradient. The tissue sections were incubated with 5 μg/ml Texas Red-conjugated EUB338 (5′-GCTGCCTCCCGTAGGAGT-3′, Sigma-Aldrich) in hybridization buffer (0.1 M Tris-HCl, 0.9 M NaCl, 0.1% SDS, and 10% formamide, pH 7.2) at 40 °C overnight. The sections were rinsed in washing buffer (20 mM Tris-HCl, 0.9 M NaCl, pH 7.4) at 40 °C for 15 min, stained with 1 μg/ml 4,6-diamidino-2-phenylindole and mounted with Prolong Gold mounting medium (Invitrogen) [21]. ISH analysis was performed as described [21]. Images were obtained and analyzed with a Nikon i80 microscope.

### Electron microscopy

Samples of the distal colon from various regimens were minced into small cubes, fixed in 4% paraformaldehyde and 2% glutaraldehyde in cacodylate buffer (0.1 M sodium cacodylate, pH 7.6) overnight at room temperature, postfixed in 1% osmium tetroxide for 90 min, dehydrated through a graded series of ethanol, embedded in epon-araldite resin, and maintained for 48 h at 60 °C to polymerize. Ultra-thin (100 nm) sections cut on a Leica UC-6 ultramicrotome were put on glow discharged 300 mesh copper grids and stained with uranyl acetate and Sato’s lead to enhance contrast. Ultra-thin sections were examined with a JEOL JEM-1400 electron microscope.

### Crypt and CLP isolation, flow cytometry and organoid assay

Crypts representing epithelial cells were isolated from distal colons as described [17–21, 27, 28]. Following crypt removal, the CLP was extracted, and both isolated crypts and CLPs were processed for biochemical and molecular assays [18]. For flow cytometry or organoid assay, isolated crypts were either stained with appropriate antibodies and data were analyzed on FACS Aria III flow cytometer (BD Biosciences) using the FlowJo software (BD Biosciences) or cultured in organoid culture media (STEMCELL Technologies Inc.) [21].

### BMDC isolation and culture and experiments with human neutrophils

To obtain BMDCs, bone marrow was isolated by flushing with RPMI with a syringe from mouse femur, cultured in RPMI-1640 media with 10% fetal bovine serum and penicillin–streptomycin, and supplemented with 20 ng/ml of granulocyte macrophages colony-stimulating factor (R&D System) for 7 days [39, 40]. Cells were infected with CR for 3 h, washed and treated with DBZ (50 nM) for 24 h, and washed, and cellular extracts were subjected to western blotting. Human neutrophils were treated with LPS (10 μg/ml) or LPS + DBZ (50 nM) for 8 h before RNA isolation and quantitative PCR (qPCR) [21].

### Histology, immunohistochemistry, and immunocytochemistry

For histology, tissues were fixed in 10% neutral buffered formalin or Carnoy’s fixative (60% methanol, 30% chloroform, and 10% acetic acid) before paraffin embedding. Images were obtained with an Eclipse i80 microscope (Nikon Instruments, Melville, NY, USA). Paraffin-embedded 5-μm-thick sections were stained with H&E for gross morphology and with Alcian blue to detect goblet cells. Immunohistochemistry and immunocytochemistry were performed with appropriate antibodies on paraffin-embedded sections [18, 21]. Antibodies used were rabbit anti-DCLK1 (1:200, ab31704) and Anti-CR (1:250, ab37056) from Abcam, Cambridge, UK; mouse anti-Notch1 NICD (1:200, clone OTI3E12) from Origene, Rockville, MD; rabbit anti-Hes1 (1:200, #11988) and mouse anti-Ki-67 (1:200, #9449) from Cell Signaling Technology, Danvers, MA; anti-CD68 (1:200, NB600-985) from Novus Biologicals (Littleton, CO, USA), Anti-Muc2 and anti-NE (1:200) Santa Cruz, Dallas, TX; Anti-CD11c and anti-F4/80 (Thermo Fisher), and anti-Ly6G (R&D, Minneapolis, MN). Antibody controls included omission of the primary antibody or detection of endogenous IgG staining with goat anti-mouse or anti-rabbit IgG (Calbiochem, San Diego, CA, USA).

### Measurement of MPO activity

MPO activity was measured in whole-colon tissue homogenates by a modification of the method of Grisham et al. [41]. Briefly, colon homogenate was sonicated in 1% hexadecyltrimethylammonium bromide buffer and centrifuged at 12,000 rpm at 4 °C for 20 min. MPO activity in the homogenates was measured in triplicate using a Fluorescent MPO Detection Kit (Fluoro MPO, Cell Technology) according to the manufacturer’s instructions.

### Patient samples

De-identified tissue sections from UC patients were procured from Children’s Mercy Hospital, Kansas City, MO through an approved Institutional Review Board according to institutional guidelines. FFPE de-identified sections (5 and 10 μm) from UC and IRAE patients that developed immune-mediated colitis (IRAEC), respectively, were obtained from The University of Kansas’ Biospecimen Repository Core Facility. For isolating RNA from the FFPE sections, we employed PureLink FFPE RNA Isolation Kit (Cat# K156002; ThermoFisher Scientific), according to the manufacturer’s instructions and the RNAs were subjected to reverse transcriptase-qPCR as described [21].

### Statistical analysis

The values are expressed as mean ± SD. Statistical analyses of all studies were performed using unpaired, two-sided Student’s *t* tests [18] or one-way analysis of variance (ANOVA) models for multiple-group comparisons. Linear contrasts with Bonferroni corrections for multiple testing were for pairwise comparisons of groups based on parameter estimates from one-way ANOVA model fits. The D’Agostino and Pearson omnibus *K*^2^ tests were used to assess the assumption of normality. *P* < 0.05 was considered statistically significant. Sample size was determined based on the results from pilot studies with similar mouse numbers. All experiments used *n* = 5 mice per group unless otherwise indicated in the figure legends and are representative of 2–3 independent experiments. For in vivo studies, reported sample number per experiment represent biologic replicates (comparison of animals of one genotype with littermates of another genotype). Both male and female mice were used in the study. All analyses were performed using GraphPad, version 9.

## Supplementary information

Supplemental Figure S1

Supplemental Figure S2

Supplemental Figure S3

Supplemental Figure S4

Supplemental Figure S5

Supplemental Table 1
