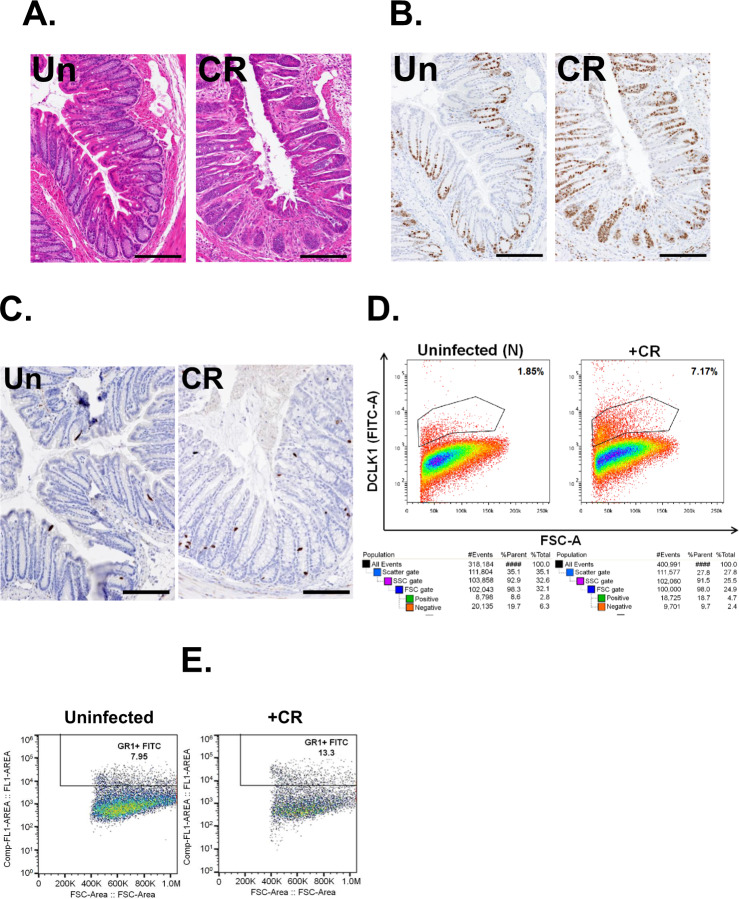# Correction: DCLK1 isoforms and aberrant Notch signaling in the regulation of human and murine colitis

**DOI:** 10.1038/s41420-021-00586-x

**Published:** 2021-08-02

**Authors:** Badal C. Roy, Ishfaq Ahmed, Jason Stubbs, Jun Zhang, Thomas Attard, Seth Septer, Danny Welch, Shrikant Anant, Venkatesh Sampath, Shahid Umar

**Affiliations:** 1 Department of Surgery, University of Kansas Medical Center, Kansas City, KS USA; 2 Department of Internal Medicine, University of Kansas Medical Center, Kansas City, KS USA; 3 Children’s Mercy Hospital, Kansas City, MO USA; 4 Children’s Hospital, Aurora, CO USA; 5 Department of Cancer Biology, University of Kansas Medical Center, Kansas City, KS USA

**Keywords:** Infection, Bacterial infection

Correction to: *Cell Death Discovery* 10.1038/s41420-021-00526-9, published online 17 June 2021

The original version of this article unfortunately contained a mistake in Figure 3. Figure 3F was in addition to Figure 5C. Figure 5C clearly defines the findings explained in the text under the heading “CR infection increases the expression of DCLK1, a tuft cell marker, in the colons of immune-incompetent mice”. Thus, Figure 3 was revised. The authors apologize for the mistake. The original article has been corrected.