# Supplementary figures and images for: DCLK1 isoforms and aberrant Notch signaling in the regulation of human and murine colitis
Source: Cell Death Discov. 2021 Jun 17;7:169. doi: 10.1038/s41420-021-00526-9 (PMC8257684; doi:10.1038/s41420-021-00526-9)

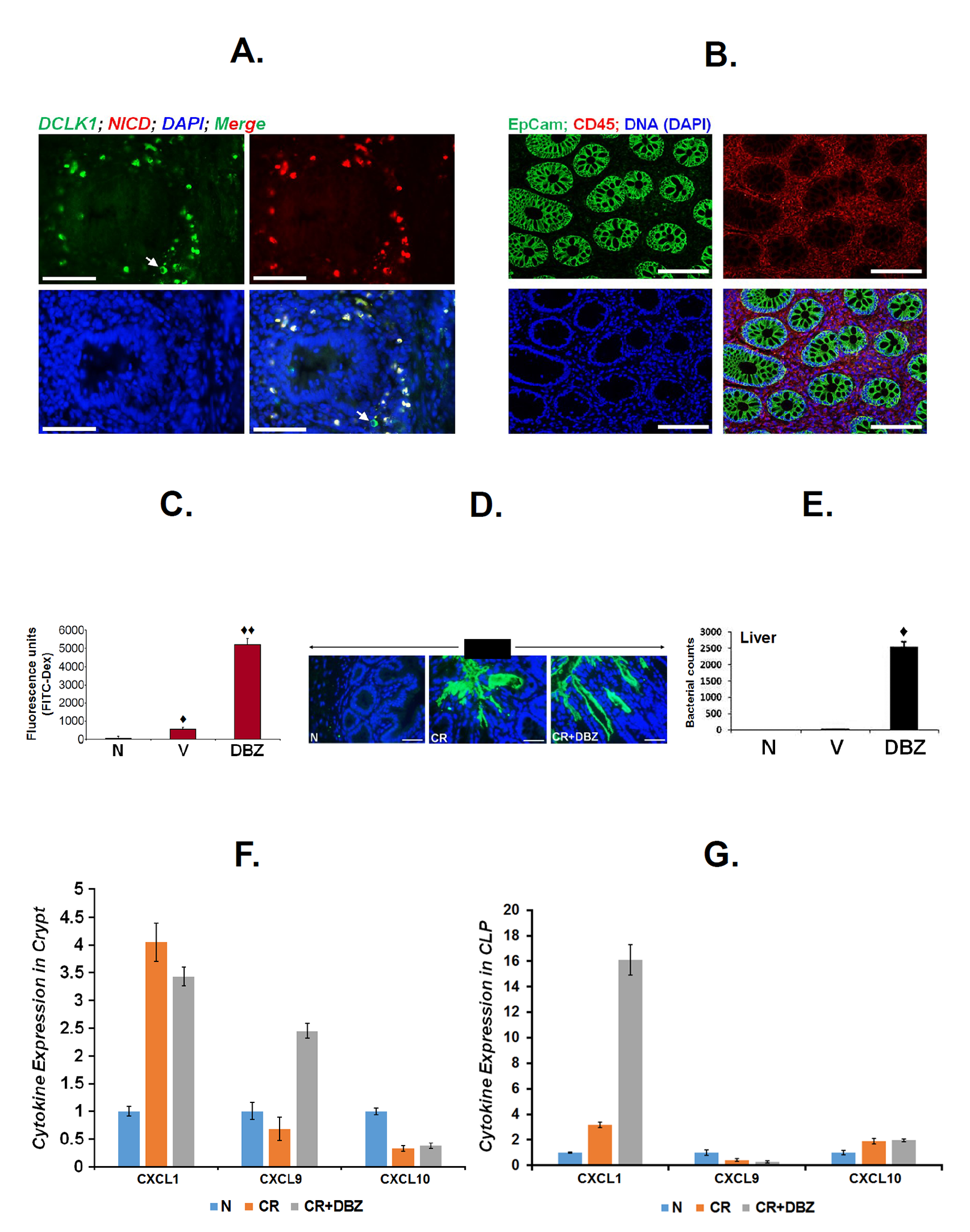

Supplement: Supplementary file 1 — Supplemental Figure S1 [file 41420_2021_526_MOESM1_ESM.tif]

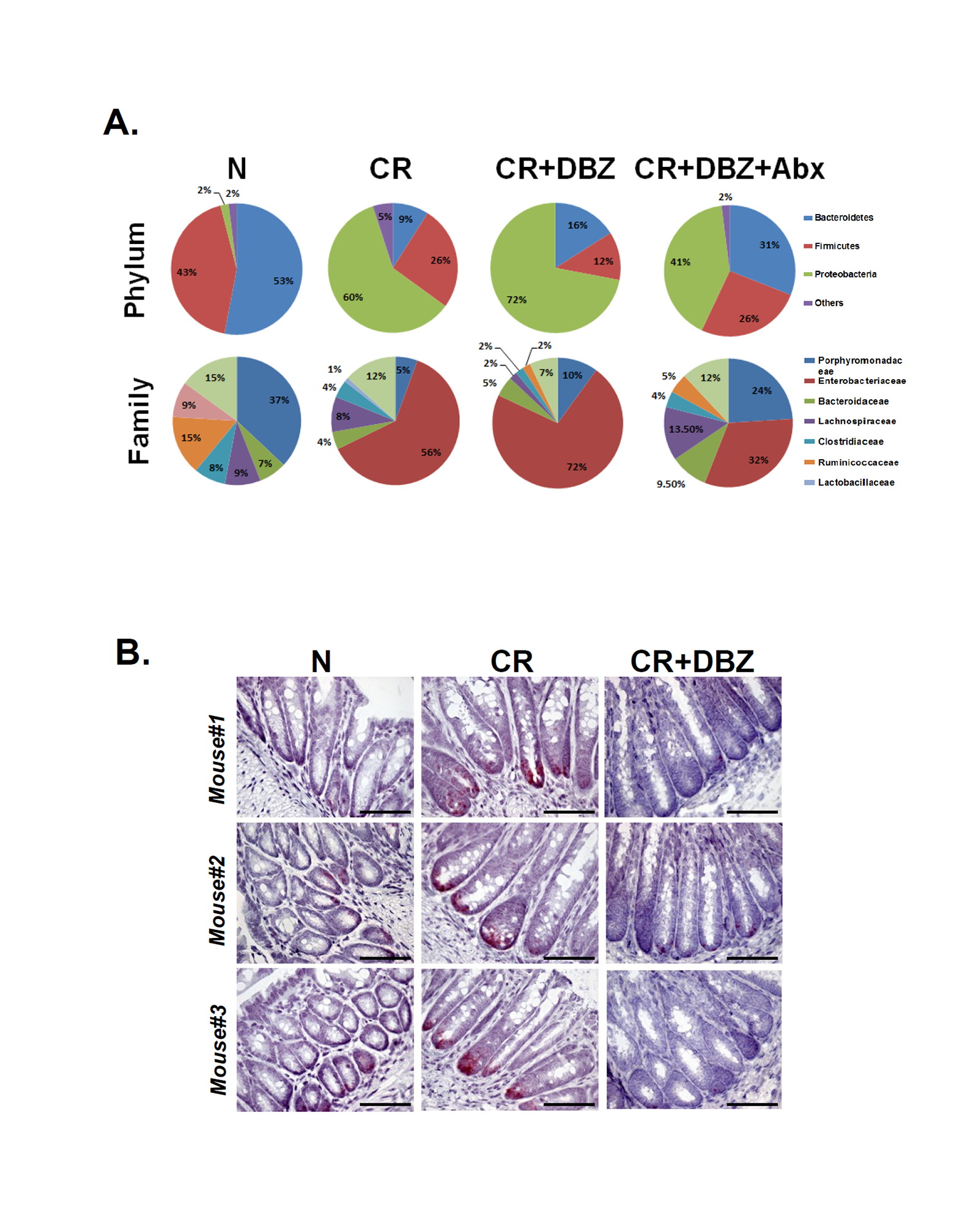

Supplement: Supplementary file 2 — Supplemental Figure S2 [file 41420_2021_526_MOESM2_ESM.tif]

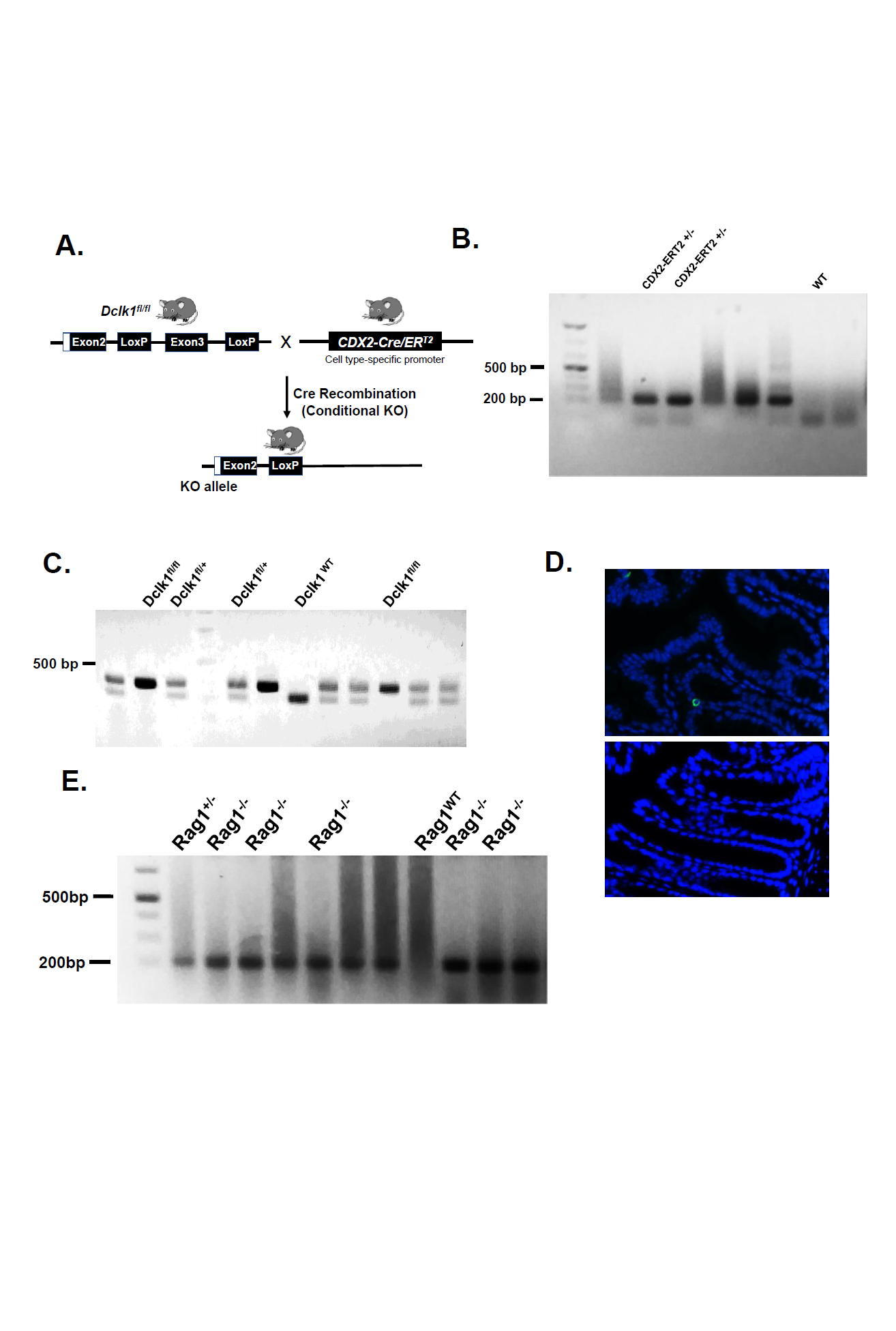

Supplement: Supplementary file 3 — Supplemental Figure S3 [file 41420_2021_526_MOESM3_ESM.tif]

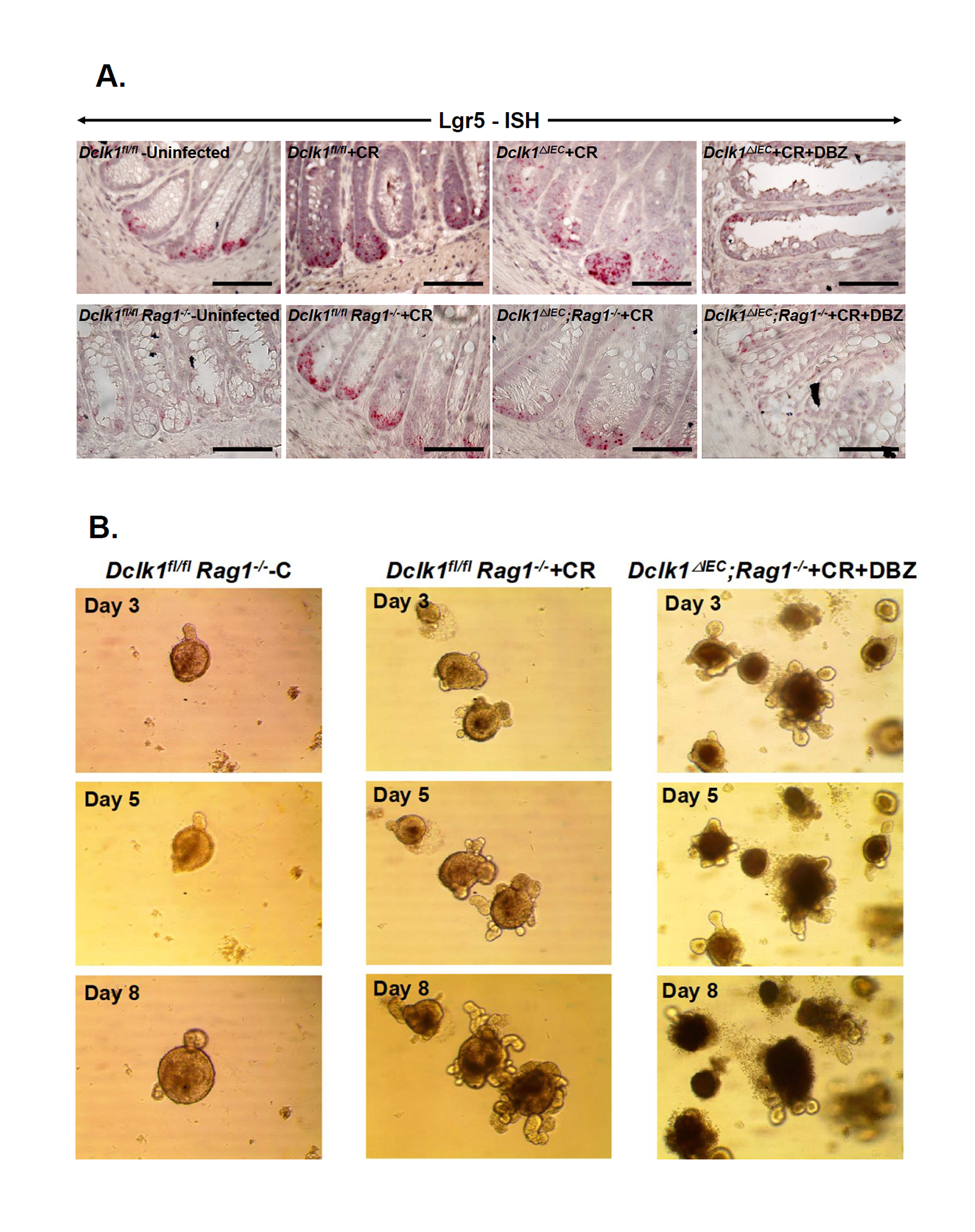

Supplement: Supplementary file 4 — Supplemental Figure S4 [file 41420_2021_526_MOESM4_ESM.tif]

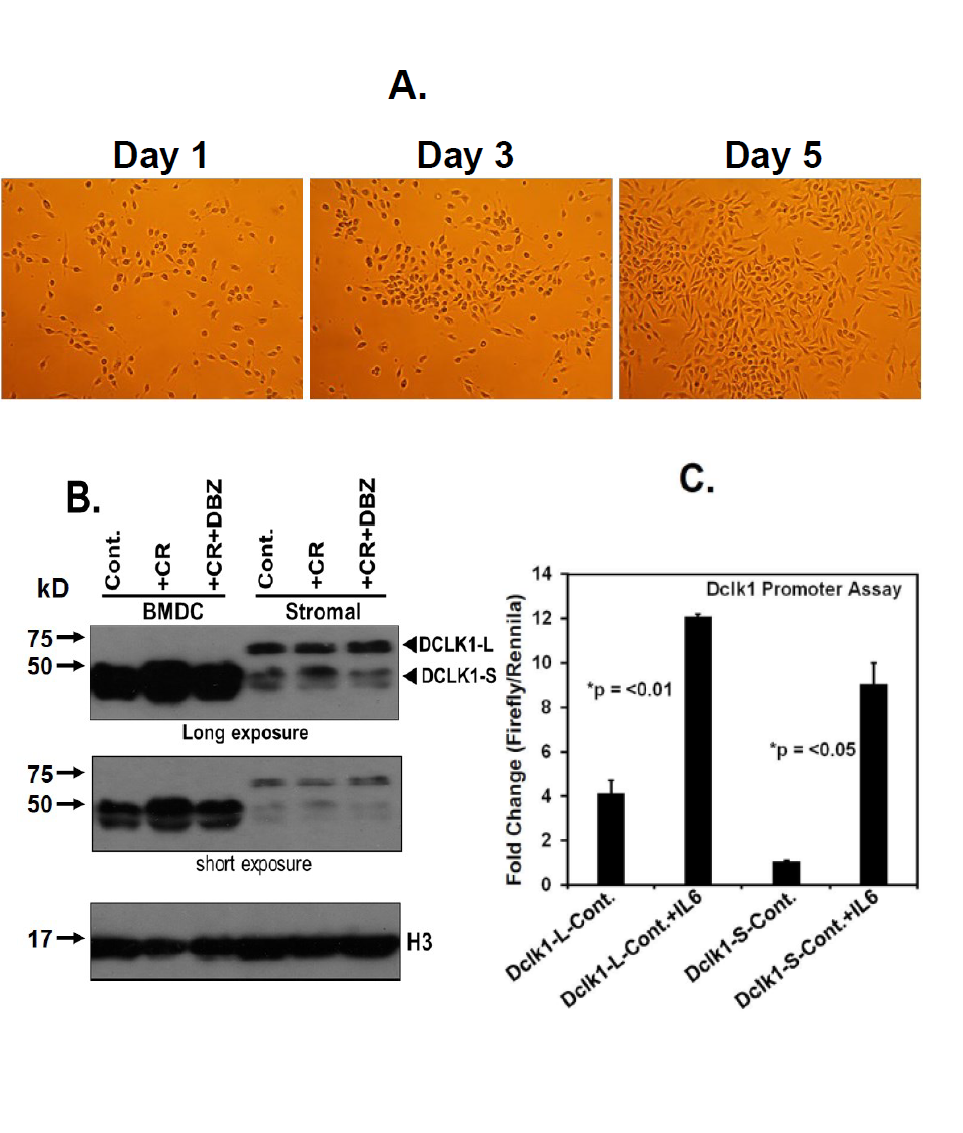

Supplement: Supplementary file 5 — Supplemental Figure S5 [file 41420_2021_526_MOESM5_ESM.tif]
