# Supplementary material for: DCLK1 isoforms and aberrant Notch signaling in the regulation of human and murine colitis
Source: Cell Death Discov. 2021 Jun 17;7:169. doi: 10.1038/s41420-021-00526-9 (PMC8257684; doi:10.1038/s41420-021-00526-9)
Supplement: Supplementary file 6 — Supplemental Table 1 [file 41420_2021_526_MOESM6_ESM.docx]

**TABLE 1. List of primers used in the study**

**Genes Forward primers Reverse primers**

Mouse Notch1 tgagactgccaaagtgttgc gtgggagacagagtgggtgt

Human Notch1 aggactgcagcgagaacatt ggggacactcgcagtagaag

Mouse Hes-1 cccacctctctcttctgacg aggcgcaatccaatatgaac

Mouse Dclk1-L tccgagaagaaagccaagaa cgaaagatctgaagcggtct

Human Dclk1-L aaacggctcattcctttgag agtcctgaaggcacatcacc

Mouse Dclk1-S gtcagccttacgcaggaaaa tgggaagcagttggattagc

Human Dclk1-S aggcatctgctgatgaatcc tctcagcactaagccaagca

Mouse NE gcactggcctcagagattgt cagaaatgacctccacgcct

Human NE aacgtctgcactctcgtgag gaaggaggcaattccgtgga

Mouse FoxD3 tgcagctacagctcaacacc tgttctcgatgctgaacgac

Human FoxD3 caaccgcttcccctactaca gggatcttgacgaagcagtc

Mouse Cxcl-1 cttgaaggtgttgccctcag tggggacaccttttagcatc

Mouse Cxcl-9 acggagatcaaacctgccta tttttccccctcttttgct

Human Cxcl-9 gagtgcaaggaaccccagta ttggggcaaattgtttaagg

Mouse Cxcl-10 gctgcaactgcatccatatc cgtggcaatgatctcaacac

Human Cxcl-10 ccccacgttttctgagacat aaggcagcaaatcagaatgg

Mouse MPO tacccccgagactttgtcag atagcacaggaaggccaatg

Human MPO aggacaaataccgcaccatc gaagagagaagccgtcctca

Mouse Notch2 cctgaacgggcagtacattt gcgtagcccttcagacactc

Human Notch2 tatatttgcacctgcccaca ttttcctgcatgctcacaag

Mouse GAPDH aactttggcattgtggaagg acacattgggggtaggaaca

Human GAPDH aggctggggctcatttgcagg tgaccttggccaggggtgct

***FOR GENOTYPING:***

Cdx2 Transgene acatgtccatcaggttcttgc aggagccagcggagcac

Dclk1 cttcccactgatatgttcattc (Mutant) agtgagatggtttacaggcaag (Common)

Rag1 tggatgtggaatgtgtgcgag (Mutant) cattccatcgcaagactcct (Common)
